# Stunting and Its Associated Factors among Early Adolescent School Girls of Gondar Town, Northwest Ethiopia: A School-Based Cross-Sectional Study

**DOI:** 10.1155/2020/8850074

**Published:** 2020-10-23

**Authors:** Azmera Tamrat, Yigizie Yeshaw, Abel Fekadu Dadi

**Affiliations:** ^1^Gondar University Referral and Specialized Hospital, Gondar, Ethiopia; ^2^Department of Medical Physiology, School of Medicine, College of Medicine and Health Science, University of Gondar, Gondar, Ethiopia; ^3^Department of Epidemiology and Biostatistics, Institute of Public Health, College of Medicine and Health Science, University of Gondar, Gondar, Ethiopia; ^4^College of Medicine and Public Health, Flinders University, SA 5001, Australia

## Abstract

**Introduction:**

Stunting is a crucial indicator of long-term chronic undernutrition that reflects a failure to reach a linear growth. Adolescent girls are potentially at a higher risk of stunting as they are traditionally married at an early age in low-income countries. In Ethiopia, stunting has mostly been examined in early childhood, with limited information at the early adolescent age. Therefore, this study is aimed at determining the prevalence of stunting and its associated factors among early adolescent school girls age 10 to 14 in Gondar town.

**Methods:**

We conducted a school-based cross-sectional study. A multistage sampling method was used to sample 662 adolescent girls in selected primary schools. A pretested, structured, and interviewer-administered questionnaire was used to collect the required data. Stata Version 14 and WHO Anthro-plus software were used to analyze the data. The bivariable and multivariable logistic regression model was fitted to identify factors associated with stunting. Adjusted Odds Ratio (AOR) with its 95% confidence interval (CI) was calculated, and a *p* value ≤ 0.05 was considered to declare statistically significant variables.

**Results:**

The prevalence of stunting was 27.5% [95% CI: 25.5%-29.5%]. The odds of stunting were found to be higher among grade 5 students [AOR; 95% CI: 1.90; 1.13-3.20], those who reported a daily meal frequency of less than three [AOR; 95% CI: 2.37; 1.60-3.50], and those who were from food-insecure families [AOR; 95% CI: 2.52; 1.70-3.73]. Adolescent girls whose mothers were government employees [AOR; 95% CI: 0.48; 0.26–0.89] or merchants [AOR; 95% CI: 0.43; 0.28–0.67] were less likely to be stunted compared to those whose mothers were housewives.

**Conclusion:**

Stunting among early adolescent girls is found to be a moderate public health problem. A school-based nutritional program might be helpful to reduce stunting in this group of adolescent girls.

## 1. Introduction

The United Nations (UN) defines early adolescence as those in the age category of 10-14 years. (1, 2). Adolescence is a period of dramatic physical, cognitive, social, and emotional changes by which 15-20% of adult height is gained. The adolescent period requires adequate nutrition to maintain the development of optimal physical, cognitive, and linear growth (3, 4).

Stunting is a crucial indicator of long-term chronic undernutrition that reflects a failure to reach a linear growth due to prolonged food deprivation and illnesses during an early stage of life (5). Adolescent girls are potentially at a higher risk of stunting as they are traditionally married and being pregnant at an early age (5, 6). Stunting can result in reduced work capacity, weaker social skills, behavioral problems, and metabolic diseases during adulthood (7–9). More importantly, through fetal programming, stunted adolescent girls are more likely to give birth to underweight infants that would be stunted in later life (6, 10).

Based on previous studies, stunting among adolescent girls is 48% in Bangladesh and 47% in Nepal (11, 12). The two Indian studies reported a stunting prevalence of 46.6% (13) and 34.2% (11) in rural adolescent girls. In Ethiopia, the prevalence of stunting among adolescent girls ranges from 26.5% to 41.8% (14–16).

Different factors are associated with the stunting of early adolescent girls. Adolescents who came from a family size of greater than five, use unimproved sources of drinking water (17), have mothers with lower levels of educational attainment, and belong to food-insecure households (18) were more likely to be stunted. Being exposed to a contaminated environment and poor hygiene (19), skipping meals in the last two weeks, and having a poor dietary diversity score are also associated with an increased risk of stunting (20).

Although adolescent stunting was a common problem in Ethiopia, limited research has been published. Therefore, this study was conducted to assess the prevalence of stunting and its associated factors among the early adolescent school girls within an urban area of northwest Ethiopia.

## 2. Methods and Materials

### 2.1. Study Design, Setting, and Population

A school-based cross-sectional study was conducted to estimate the prevalence of stunting and its associated factors among early adolescent girls aged between 10 and 14 years and attending a government primary school in Gondar town. The data collection was conducted in January 2016. Gondar town is located in the Northwest region of Ethiopia, about 735 km from Addis Ababa, the capital city of Ethiopia. In the town, there are forty-three public primary schools and 16,331 students, of which 8,885 are early adolescent girls. All early adolescent girls present in selected governmental primary schools at the time of data collection were included. Those with a physical disability and critical illness were excluded.

### 2.2. Sample Size and Sampling Procedure

The sample size was calculated using a single population proportion formula by considering the following assumptions: stunting among adolescent girls in Debre Markos Town, Amhara region (*p* = 0.315) (17), a 5% margin of error, a 95% confidence level, a nonresponse rate of 10%, and a design effect of 2. Accordingly, the final sample size was 731. In the first stage, 10 primary schools were selected from 43 primary schools using a lottery method (23% of all primary schools). In the second stage, the total number of adolescent girls was proportionally allocated according to the size of the primary schools, and the final study participants were selected from each included primary school using a simple random sampling.

### 2.3. Variables of the Study

The dependent variable in this study was early adolescent stunting. Early adolescent stunting is defined by a height for age *Z*-score (HAZ) of less than minus two standard deviations (-2 SD) from the median value of the WHO reference standard. Participants with HAZ scores of less than minus three standard deviations were severely stunted. The independent variables were education level of the student, educational status of the family, income, employment status, family size, information about food types such as teff (a type of crop used to make Enjera, a traditional food in Ethiopia), daily meal frequency, source of water, hand washing habits, and food security status. Food security was categorized as food secure for households with a raw score of 0–1, and food insecure for households with a raw score of 2 and above (22). The Household Food Insecurity Access Scale was used to collect and categorize food security, and it has been validated in Ethiopia.

### 2.4. Data Collection Procedures and Quality

Data were collected from the students using a structured and interviewer-administered questionnaire. The questionnaire is prepared in English first and translated to Amharic and then retranslated to English to check for its consistency. The weight of adolescent girls was measured to the nearest 0.1 kg using a calibrated SECA weighing scale with light clothing and no shoes. The height of adolescent girls was also measured to the nearest 0.1 cm on a standing position without shoes using the same weighing device fitted with a sliding headpiece. Four diploma nurse data collectors and one Bachelor of Science nurse supervisor were recruited and trained for data collection. The questionnaire was pretested, and unclear questions were revised accordingly to maintain data quality. The weighing scale was zeroed before taking every other measurement. The collected data were reviewed and checked for completeness before data entry.

### 2.5. Data Processing and Analysis

After checking the collected data for completeness, it was entered into Epi-data version 3.1 and exported to Stata version 14.0 for analysis. The anthropometric measurement of height-for-age was calculated using the WHO Anthro-plus software. Bivariable and multivariable logistic regression analyses were performed to assess the association between different explanatory variables and early adolescent stunting. All variables with a *p* value < 0.2 in the bivariable analysis were entered into the multivariable logistic regression model. Odds ratio with its 95% confidence intervals was estimated to identify factors associated with stunting. A *p* value ≤ 0.05 was considered to be statistically significant. Model fitness was checked using the *Hosmer* and *Lemeshow* goodness of fit test (*p* value = 0.99).

### 2.6. Ethical Consideration

Ethical clearance of this study was granted by an Ethical Review Board of the University of Gondar. Permission was also obtained from the Gondar town Education Office and selected schools. Written consent from the guardians and assent from each study participant was taken before data collection. The information collected from this study remained confidential. Those who were severely stunted offered dietary counseling and linked to the nearest health institution for further management.

## 3. Results

### 3.1. Sociodemographic Characteristics of the Respondents

Six hundred and sixty-two (662) study participants were included in the study with a response rate of 90.56%. The mean age and standard deviation (±SD) of the study participants were 12.85 (±1.23) years. Most of 545 (82.3%) study participants were Orthodox Christian. Among the respondents, 388 (58.6%) had a family size of five and above. Four hundred and eighty-one (72.7%) of the study participants had alive family (both the mother and father are alive), and 182 (27.5%) of the study participants were grade seven (Table 1).

### 3.2. Nutrition and Health-Related Variables of the Respondents

From a total of 662 early adolescent school girls, 402 (60.7%) of them had a daily meal frequency of three and above. Three hundred and twenty-two (48.6%) of the study participants were identified as being food insecure. Regarding the availability of pure water, 34 (5.1%) of the study participants used river water. One hundred and twenty-nine (19%) of the participants reported an episode of diarrhea within the last two weeks. Four hundred and seventy-one (71.1%) of participants had got information about food types from different sources (media, health professionals, and schools) (Table 2).

### 3.3. Prevalence and Associated Factors of Stunting

The overall prevalence of stunting in this study was 27.5% (95% CI: 25.5%-29.5%). Of the total study participants, 60 (9.1%) were found to be severely stunted. On bivariable logistic regression analysis, stunting was associated with the grade level of students, education level of their parents, employment status of their parents, whether their family is alive or not, the frequency of meals consumed per day, food security status, and with whom they are living with. However, after adjusting for other covariates, the grade level of students, the employment status of their mother, frequency of meals consumed per day, and food security status were significantly associated with stunting (**p** ≤ 0.05).

The odds of stunting among grade five students were 1.9 times higher than those among grade eight students [95% CI: 1.13-3.20]. Students whose mothers were employed had a 57% reduced chance of being stunted than those whose mothers were housewives [AOR = 0.43; 95% CI: 0.28-0.67]. Students who had a merchant mother had a 52% reduced chance of being stunted compared with those whose mother was a housewife [AOR = 0.48, 95% CI: 0.26-0.89]. The odds of stunting among students who had a meal frequency of less than three times per day were 2.37 times higher than those who had a meal frequency of three and above times per day [95% CI: 1.60-3.50]. Students who were living in food-insecure households had a 2.52 times higher chance of being stunted than those who were living in food-secure households [95% CI: 1.70-3.73] (Table 3).

## 4. Discussion

This study tried to determine the prevalence of stunting and associated factors among early adolescent school girls. Grade level of students, employment status of their mother, frequency of meals consumed per day, and food security status were significantly associated with stunting among early adolescent school girls.

The prevalence of stunting in this study was 27.5%, which is similar to a study conducted in Tigray (26.5%) (15). This might be due to similarities in sociocultural conditions, feeding practices, economic growth, and level of awareness about the importance of proper nutrition during adolescents between the two regions of Ethiopia. However, it is higher than the one conducted in Addis Ababa (7.2%) (21) and a report by the Ethiopian Health and Nutrition Research Institute (EHNRI) (23%) (23). This difference could be related to variations in the level of awareness among Addis Ababa and Gondar town mothers. The importance of proper feeding practice and attention given for adolescent girls' nutrition by their families could be different in Addis Ababa than Gondar town. Moreover, economics influencing access to health care and availability of high quality foods generally play a key role in influencing differences between groups in the level of stunting. Furthermore, the difference in prevalence between our findings and the findings from EHNRI might be related to the size of the study. While our study was confined to a single town, the EHNRI study involved a nationally representative sample.

Our estimate was lower than an estimate reported by a study conducted in adolescent girls of Amhara region (31.5%) (17), Bangladesh (49%) (24), and India (50.3%) (13) and early adolescent school girls of India (34.2%) (14), Nepal (47%), and Myanmar (39%) (11). These variations might be explained by the differences in a study setting, economic status, sociocultural conditions, data collection instruments, and study populations.

The odds of stunting among grade five students were 2 times higher than those among grade eight students. This finding was consistent with a study conducted in Jimma zone (25). This might be due to the increased level of nutrition awareness of grade eight students than students in a lower grade. Participants who had a meal frequency of three and more times per day had reduced odds of stunting than those reporting a lower frequency, which is in line with a study conducted in the Oromia region (21). Adequate meal frequency indeed accelerates a linear growth of adolescents by sufficiently supplying essential nutrients for their body size. Adolescents who lived in food-insecure households had 2.5 times higher odds of being stunted than those who lived in food-secure households. This finding is in line with a study conducted in the Amhara region and Dale Woreda (16, 19). This could be true as food is very vital for the physical growth of adolescents in addition to its health benefits. Participants born from mothers who were government employees or merchants had a reduced chance of being stunted than those born from a housewife. Ethiopia is one of the patriarchal countries, and housewife mothers are waiting for the hand of their husbands to feed their kids as compared to those who independently fetch their income. Furthermore, housewife mothers are more likely to be uneducated and have low job access and decision-making power in the household, which would affect their knowledge about nutrition and the ability to earn a better income to care for their children (26).

As a limitation, there is a potential for recall bias, especially concerning food frequency questions. Another limitation of this study might be the social desirability bias. Moreover, since the study is a cross-sectional study, it also shares the limitations of cross-sectional study design, of which the issue of temporality is one. Hence, we are unable to show weather the outcome or some other independent variables come first. Therefore, the interpretation of these findings should consider these inherence limitations. However, our study clearly showed that adolescent nutritional interventions have been lagging in Ethiopia (27) though adolescent is the best window to intervene such nutritional problems (28). We hoped the recent National Nutrition (NNP) Program and the National School-based Nutrition Program (NSBNP) would bring a significant change in such burden of early adolescent stunting (29).

## 5. Conclusion

The finding of this study demonstrated that stunting among early adolescent girls is a moderate public health problem. Being in a lower grade, having housewife mothers, daily meal frequency of less than three, and living in the food-insecure household increased the odds of being stunted. It is crucial to improve the food security status of households by increasing their income. It is also vital to increase nutrition awareness of the family and adolescent girls to improve their nutritional intake and tackle the intergenerational effect of adolescent malnutrition.

## Figures and Tables

**Table 1:** Sociodemographic characteristics of early adolescent school girls, Gondar town, Northwest Ethiopia, 2016.

Variable/category	Number	Percentage
Age (in years)		
10	38	5.8
11	73	11.0
12	112	16.9
13	166	25.1
14	273	41.2
Religion		
Orthodox	545	82.3
Muslim	69	10.4
Protestant	45	6.8
Catholic	3	0.5
Grade of students		
Grade 5	178	26.9
Grade 6	173	26.1
Grade 7	182	27.5
Grade 8	129	19.5
Mother's education status		
Illiterate	148	22.3
Read & write	244	36.9
Diploma	162	24.5
Degree and above	108	16.3
Father's education status		
Illiterate	68	10.3
Read & write	221	33.4
Diploma	175	26.4
Degree and above	198	29.9
Father's occupation		
Farmer	89	13.4
Craftsman	121	18.3
Merchant	154	23.3
Government employee	292	44.1
Daily laborer and private servant	6	0.9
Mother's occupation		
Housewife	292	44.3
Government employee	242	36.6
Merchant	82	12.4
Daily laborer and private servant	45	6.8
Alive parents		
Both alive	481	72.7
Mother alive	104	15.7
Father alive	44	6.6
Both dead	33	5.0
Currently live with		
Both parents	384	58
One of their parents	278	42
Family size		
Less than five	274	41.4
Five and above	388	58.6

**Table 2:** Nutrition and health-related characteristics of early adolescent school girls, Gondar town, North West Ethiopia, 2016.

Variable/category	Number	Percentage
Consume meat		
Eat < 1 per week	458	69.2
Eat ≥ 1 per week	204	30.8
Consume milk		
<1 in week	529	79.9
≥1 a week	133	20.1
Meal frequency		
<3 meals/day	260	39.3
≥3 meals/day	402	60.7
Usual food type ingredient		
Teff (Ethiopian staple food)	563	85.0
Wheat	51	7.7
Other (maize, rice, barley)	48	7.3
HH food security status		
Secure	340	51.4
Insecure	322	48.6
Water source		
Pipe water	628	94.9
River water	34	5.1
Bed net use		
Yes	260	39.3
No	402	60.7
History of diarrhea disease		
Yes	129	19.5
No	533	80.5
Have information about food types		
Yes	471	71.1
No	191	28.9
Hand wash after toilet		
Yes	601	90.8
No	61	9.2

**Table 3:** Bivariable and multivariable logistic regression analysis on factors associated with stunting among early adolescent school girls in Gondar town, Northwest Ethiopia, 2016.

Variables	Stunting		Odds ratio	
	Yes, *N* (%)	No, *N* (%)	COR (95% CI)	AOR (95% CI)
Educational status of mother				
Illiterate	51 (35.4%)	93 (64.6%)	2.40 (1.31-4.40)	0.59 (0.35-1.01)
Read & write	80 (34.0%)	155 (66.0%)	2.26 (1.30-3.97)	0.69 (0.42-1.12)
Diploma	32(17.7%)	149 (82.3%)	0.94 (0.50-1.80)	0.55 (0.30-1.04)
≥Degree	19 (18.6%)	83 (81.4%)	1	1
Grade of students				
Grade 5	65 (36.5%)	113 (63.5%)	1.80 (1.25-2.61)	1.90 (1.13-3.20)^∗^
Grade 6	37 (21.4%)	136 (78.6%)	0.65 (0.43-0.97)	1.32 (0.72-2.44)
Grade 7	54 (29.7%)	128 (70.3%)	0.61 (0.38-0.98)	1.27 (0.68-2.56)
Grade 8	26 (20.2%)	103 (79.8%)	1	1
Eating frequency/day				
<3 per day	111 (42.7%)	149 (57.3%)	3.47 (2.43-4.95)	2.37 (1.60-3.50)^∗^
≥3 per day	71 (17.7%)	331 (82.3%)	1	1
Household food security				
Food insecure	129 (40.1%)	193 (59.9%)	3.62 (2.51-5.23)	2.52 (1.70-3.73)^∗^
Food secure	53 (15.6%)	287 (84.4%)	1	1
Live with				
Both mother & father	98 (25.5%)	286 (74.5%)	1	1
With father only	46 (29.1%)	112 (70.9%)	1.15 (0.57-2.20)	0.88 (0.37-2.11)
With mother only	13 (27.7%)	34 (72.3%)	0.90 (0.79-1.81)	1.10 (0.60-1.84)
Others^b^	25 (34.2%)	48 (65.8%)	1.52 (0.89-2.59)	1.20 (0.63-2.27)
Alive parents				
Both mother & father	128 (26.6%)	353 (73.4%)	1	1
One of their parents alive	54 (29.8%)	127 (70.2%)	1.20 (0.80-1.71)	0.91 (0.58-1.40)
Mother employment status				
Housewife	116 (39.6%)	177 (60.4%)	1	1
Government employee	40 (16.5%)	202 (83.5%)	0.30 (0.20-046)	0.43 (0.28-0.67)^∗^
Merchant	16 (19.5%)	66 (80.5%)	0.37 (0.20-0.67)	0.48 (0.26-0.89^∗^
Other^a^	10 (22.2%)	35 (77.8%)	0.43 (0.21-0.91)	0.40 (0.18-0.87)^∗^

^∗^Statistical significant (*p* value < 0.05). ^a^Daily laborer and private servants. ^b^Sister, brother, aunt, and uncle.

## Data Availability

The data upon which the result was based could be accessed by a reasonable request made to the corresponding author.

## Abbreviations

AOR: Adjusted Odds Ratio
COR: Crud Odds Ratio
UN: United Nations
EHNRI: Ethiopian Health and Nutrition Research Institute
NNP: National Nutrition Program
NSBNP: National School-based Nutrition Program
WHO: World Health Organization.
